# Clinical and Neuropsychological Predictors of Posttraumatic Stress Disorder

**DOI:** 10.1097/MD.0000000000000113

**Published:** 2014-11-07

**Authors:** Sharain Suliman, Dan J. Stein, Soraya Seedat

**Affiliations:** MRC Anxiety Disorders Unit (S Suliman, DJS, S Seedat), Department of Psychiatry, Stellenbosch University; Department of Psychiatry and Mental Health (DJS), University of Cape Town; and Department of Psychiatry (S Seedat), Stellenbosch University, Cape Town, South Africa.

## Abstract

Although acute responses to traumatic stress generally resolve within a few weeks, some individuals experience severe and persistent problems, such as posttraumatic stress disorder (PTSD). While studies have identified a variety of predictors of PTSD, not all data are consistent. This longitudinal study examined the predictive power of neurocognitive deficits with regard to PTSD severity.

One hundred thirty one road traffic collision (RTC) survivors were included within 2 weeks of the RTC and followed up 3 and 6 months later to determine severity of PTSD.

Impairment on tests of information processing, executive functioning, verbal learning, and motor speed predicted PTSD severity when neuropsychological, clinical, and sociodemographic factors were all taken into account. Clinical variables (initial symptoms, psychiatric diagnoses, disability, trait anxiety, perceived stress, negative cognitions, and sleep) were associated with 3 and 6-month PTSD severity, but only trait anxiety was predictive of PTSD severity. Ethnicity and education were also found to be predictive.

These findings suggest implementation of a holistic approach to screening for PTSD and support a need for interventions that target neurocognitive, clinical, and social variables. Early targeted profiling of this group of trauma survivors can inform early clinical interventions and policy.

## INTRODUCTION

There is accumulating evidence that early intervention strategies are useful in preventing posttraumatic stress disorder (PTSD), with some studies suggesting that there is a window of brain plasticity during the acute trauma response that presents an opportunity to affect the final outcome.^1^ Two meta-analyses and several reviews of PTSD risk factors have identified key factors influencing PTSD vulnerability. These include pre-trauma factors such as family psychiatric history, prior psychological adjustment, previous trauma exposure, characteristics of the traumatic experience (namely, trauma severity), peritraumatic emotional reactions (namely, dissociation), and post-trauma social support.^2–5^ However, Brewin et al^2^ cautioned against attempts to build a general vulnerability model for all cases of PTSD based in their findings, as they found that there were substantial sectors of the population for whom gender, age at trauma, and race could not be shown to be risk factors at all, indicating that other identifiers are needed.

There have been relatively few prospective studies of PTSD that have measured risk factors before the traumatic event,^2^ although there have been some that have measured risk factors post-trauma but prior to the onset of PTSD. Due, in part, to methodological limitations of extant research, the role of neuropsychological factors and structural and functional changes of the brain in the evolution of PTSD are also less well understood.^6^ Neuropsychological dysfunction in both acute and chronic PTSD includes disturbances of learning, memory, attention, concentration, and executive functions.^7–10^ Whether these deficits are precursors to or a consequence of PTSD, or risk factors present prior to or early on in the trauma response, is unclear.

Only 3 published studies have prospectively assessed for cognitive deficits. These have implicated verbal memory, sustained attention, verbal learning, visuospatial memory, and reaction-time proficiency deficits, and trends toward poorer cumulative learning and verbal fluency in the prediction of PTSD severity.^11–13^ Educational achievement pre-trauma and lower intelligence quotient (IQ) (albeit within the normal range) have also been reported to be associated with PTSD symptoms.^14,15^ Studies in twin pairs have further highlighted that poorer performance on certain neurocognitive tests may indicate a vulnerability factor for developing symptoms of PTSD rather than solely representing an outcome of PTSD symptoms.^16,17^ Taken together, these studies suggest that neurocognitive capacities may serve as premorbid risk or protective factors in PTSD and may relate to processes that determine the resolution versus the maintenance of early posttraumatic reactions.

The aims of this study were to extend existing knowledge of early neuropsychological predictors of PTSD. The primary aim was to examine the predictive power of neurocognitive deficits with regard to PTSD severity, while taking potentially confounding clinical, road traffic collision (RTC)-related, and sociodemographic variables into account. The secondary aim was to determine if neurocognitive performance and psychiatric symptoms, such as those associated with anxiety, disability, and acute stress disorder (ASD) at 2 weeks post-trauma, were associated with severity of PTSD symptoms. We hypothesized that worse early neurocognitive performance and clinical symptoms would significantly predict short-term (3 months) and long-term (6 months) outcome.

## METHODS

### Design

This was a prospective study of risk factors that could potentially predict the development of PTSD severity, based on the Clinician-Administered PTSD Scale (CAPS^18^) score, in a sample of RTC survivors.

### Procedures

Ethics approval for the study was obtained from the Health Research Ethics Committee, Stellenbosch University, and the hospital departments where recruitment took place (in the Cape Town region of South Africa). The participants were assessed at baseline (approximately 10 days post-RTC), using clinical and neurocognitive measures and followed up 3 and 6 months later to assess for PTSD severity. Assessments were conducted by researchers trained in all measures and written informed consent was obtained from all participants prior to study inclusion.

### Sample

One hundred thirty-eight RTC survivors, who were involved in RTCs as drivers, passengers, or pedestrians, were recruited. The participants were eligible for the study if they were willing and able to provide written informed consent, between the ages of 18 and 65 years, able to read and write in English at 5th grade level. In order to avoid interference with participation in the study and potential confounding variables, participants were excluded if they had a current or past history of psychosis; any significant recent or previous head injury (defined as loss of consciousness for >24 hours and/or posttraumatic amnesia and/or traumatic brain injury); use of any psychotropic medication at the initial assessment; serious physical injury or threat to life at inclusion (as determined by the Abbreviated Injury Scale^19^ and chart records, ie, no serious threat to life), or where injury sequelae would interfere with study participation according to the judgment of the investigator; and/or cognitive impairment. Because of missing data, the final sample consisted of 131 participants.

### Measures

A demographic questionnaire was used to collect information on age, gender, education, employment, household income, whether the participant was the breadwinner, marital status, living arrangements, ethnicity (whether the participant was of white, black, Indian, or mixed race/colored ancestry), and past and current medical and psychiatric history.

#### Clinical Measures

The clinical measures are as follows.

1. The CAPS^18^ was our main outcome measure. It provides current and lifetime PTSD diagnostic information in addition to frequency and intensity scores of individual PTSD symptoms.
2. The MINI International Neuropsychiatric Interview (MINI^20^) is a clinician-administered, structured diagnostic interview for major psychiatric disorders based on Diagnostic and Statistical Manual (DSM)-IV diagnostic criteria, and was used to determine past and current psychiatric disorders in the sample.
3. The Acute Stress Disorder Scale (ASDS^21^) is a self-report inventory, based on DSM-IV criteria for ASD.
4. The Spielberger State-Trait Anxiety Inventory-Trait Version (STAI-T^22^) is a self-rated scale that measures current anxiety symptoms. The trait version measures a more general and longstanding anxiety, as opposed to temporary state anxiety.
5. The Perceived Stress Scale (PSS^23^) is a measure of the degree to which situations in one’s life are appraised as stressful. Items are designed to tap how unpredictable, uncontrollable, and overloaded respondents find their lives.
6. The Connor–Davidson Resilience Scale (CD-RISC^24^) is a self-report measure that assesses stress-coping ability.
7. The Sheehan Disability Scale (SDS^25^) asks respondents to rate the amount of disability caused by a specific disorder in the domains of work, home management, social life, and close relationships. Domain scores are added up to provide a total score.
8. The Posttraumatic Cognitions Inventory (PTCI^26^) assesses trauma-related cognitions in 3 domains, negative cognitions about self, negative cognitions about the world, and self-blame, which are added up to provide a total score.
9. The Pittsburg Sleep Quality Index (PSQI^27^) indicates sleep quality in 7 domains that are then added to yield 1 global score.

#### Neuropsychological Measures

The neuropsychological measures are as follows.

1. The Wechsler Abbreviated Scale of Intelligence^28^ is a short and reliable measure of intelligence that can be used in clinical, psychoeducational, and research settings. The 2 subtest form, consisting of a vocabulary and matrix reasoning test, was used to assess cognitive ability of the participants.
2. Trail Making Test (TMT^29^) is a 2-part test for memory, speed of attention, sequencing, mental flexibility, visual search, and motor and executive function. Part A was used as a measure of information processing and Part B of executive functioning.
3. The Wechsler Adult Intelligence Scale-III^30^ is a measure of general intellectual function in older adolescents and adults, in which 3 subtests were used:
  –Digit span (DS) is a 2-part test that assesses auditory attention, concentration, memory for digits (digits forward) and working memory (digits backward).
  Digit symbol/coding (DSy) is a test of psychomotor performance, visual-motor coordination, sustained attention, and motor and mental speed.
  Symbol Search (SS) assesses for attention, visual perception, and response speed.
4. The Stroop Test^31^ is a measure of selective attention and cognitive flexibility.
5. The Rey Auditory Verbal Learning Test (RAVLT^32^) measures new learning and verbal memory (immediate recall, short and long-term retention of information).
6. The Wechsler Memory Scale-R^33^ visual reproduction (VR) subtest is a task of immediate and delayed visual memory.
7. The Tower of London (ToL)^34^ tests for the ability to plan ahead/executive functioning. The total move score was used as a measure of executive planning.
8. The Wechsler Intelligence Scale for Children-Mazes^35^ test yields data about the highest levels of mental functioning involving planning and foresight, and is sensitive to executive disorders. Although designed for children, they give a rough estimate of adult performance.
9. Grooved Pegboard Test (GPT^36^) is a test of manual dexterity, complex coordination, and fine motor skill.

### Data Analyses

Statistica 12 (StatSoft Inc, Tulsa, OK, 2013) was used to analyze the data, with no replacement of missing data. All data available at a particular time point were used for the analyses. We conducted reliability analyses of the clinical measures, all of which were within acceptable limits, with Cronbach alpha values ranging from 0.62 to 0.95. Severity of PTSD at 3 and 6 months were the primary outcome measures. Univariate analyses (correlations, analysis of variance) were first conducted for all neuropsychological and clinical predictors and any potential confounders (ie, demographic, social, and RTC-related factors). In order to identify the variables that were most predictive of 3 and 6-month PTSD severity, all clinical and neuropsychological variables, and any confounding variable that emerged as significant or showed a trend toward significance (*P* < 0.10), were entered into a best subsets regression analysis. This method compares all possible models using a specified set of predictors and displays the best-fitting models possible allowing for a choice of the optimal model.^37^ Tolerance statistics determined that there were no problems of multicollinearity.

## RESULTS

### Demographic Characteristics

One hundred thirty-one participants were included in the study. Of these, 104 (79.5%) returned for the 3-month follow-up and 101 (77.1%) for the 6-month follow-up. Baseline characteristics did not differ between the full sample and the participants who returned for their 3 and 6-month follow-up visits (*P* > 0.05).

The majority of the sample was male (56.5%), of colored ethnicity (44.3%), married (29.8%), or living with a partner (35.1%), family member(s) (51.8%), or a significant other (26.6%). The average age of the sample was 33.05 (±10.54) years. Participants had a mean of 10.02 (±2.75) years of education; most were employed (77.9%) and breadwinners (61.8%), but only 39.7% had household incomes of more than ± $10,000 per annum.

### Injury Characteristics

The majority of participants were passengers in a car (28.2%) or pedestrians (28.2%). RTCs took place, on average, 9.98 (±4.78) days prior to the initial assessment. Based on the Abbreviated Injury Scale, approximately half the sample (52.7%) had minor injuries (a score of 1) while the remainder had moderate injuries (a score of 2).

### Medical and Psychiatric History

Previous medical history (ie, heart problems, tuberculosis) was reported in 41.2%, and 76.7% had been prescribed medication to treat either a medical condition or injury resulting from the RTC; 17.6% reported previous psychiatric history (ie, depression or anxiety) and 13.7% had been prescribed psychiatric medication in the past. At baseline, current major depressive disorder (18.5%) and alcohol abuse (16.9%) were the most common diagnoses. The participants had an average of 3.78 (±2.41) traumas prior to the accident, with the most common lifetime traumas being assault with a weapon (47.3%) and prior involvement in an RTC (45.0%).

### Neuropsychological Predictors

Descriptive statistics and the relationship of PTSD severity to individual neuropsychological measures are presented in Table 1.

**TABLE 1:**
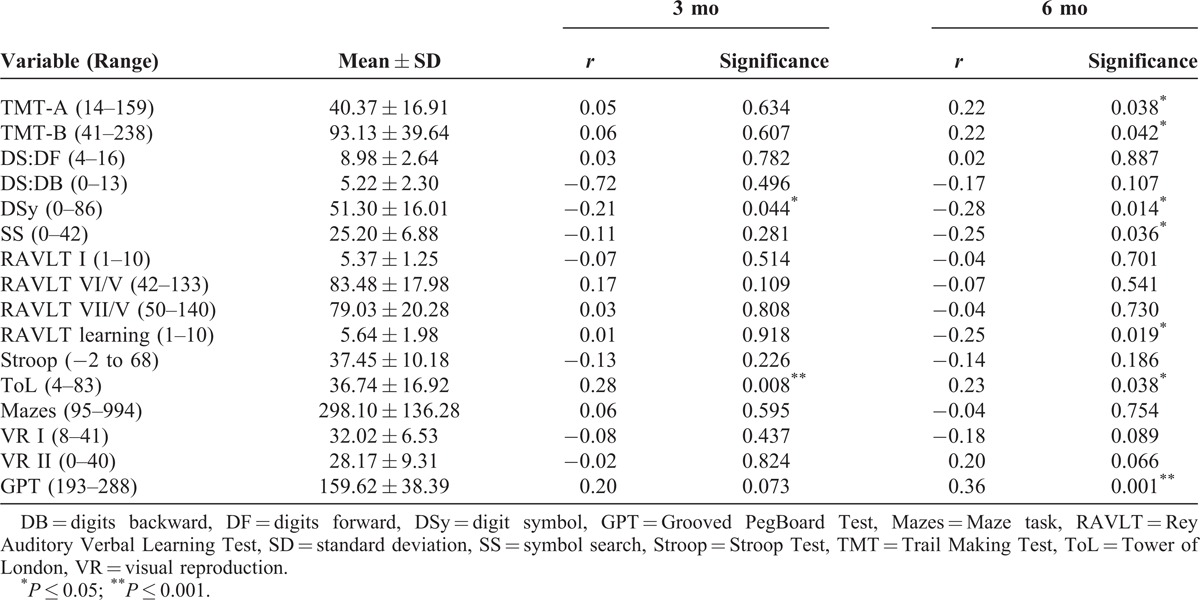
Neuropsychological Predictor Variables and Their Relationship to PTSD Severity at 3 and 6 mo

DSy and ToL scores were significantly associated with PTSD severity 3 months after the trauma, and the TMT (A) and (B), DSy, SS, RAVLT learning score, ToL, and the GPT were significantly associated with PTSD severity at 6 months post-trauma. DSy, SS, and RAVLT learning were negatively correlated so that those with more severe PTSD performed worse in these tasks, and ToL, GPT, and TMT (A) and (B) were positively correlated indicating that those with more severe symptoms took longer or made more moves in completing these tasks.

Confounding variables (those that previous research has indicated are associated with neurocognitive outcomes) that were significantly associated with any of the neuropsychological test scores (*P* < 0.05), or that showed a trend toward significance (*P* < 0.10), were included in the regression analyses. As income and education were significantly correlated (*r* = 0.52, *P* < 0.001), we excluded income as a confounder. Variables that were included were age, education, ethnicity, medication use at the time of the accident, and IQ.

### Clinical Predictors

Baseline clinical characteristics of the sample and their relationships to 3 and 6-month PTSD severity are displayed in Table 2. There were no significant differences present between participants who did and did not return for follow-up visits (*P* > 0.05). The mean CAPS score at baseline was 33.35 ± 21.08, and 30 participants (22.9%) met criteria for PTSD using a cutoff of 50, excluding the time criterion. The mean CAPS score at the 3-month visit was 29.57 ± 19.49 with 20 participants (19.6%) meeting criteria for a diagnosis of PTSD. This number dropped to 22.11 ± 17.22, with 12 participants (12.2%) meeting criteria at the 6-month visit.

**TABLE 2:**
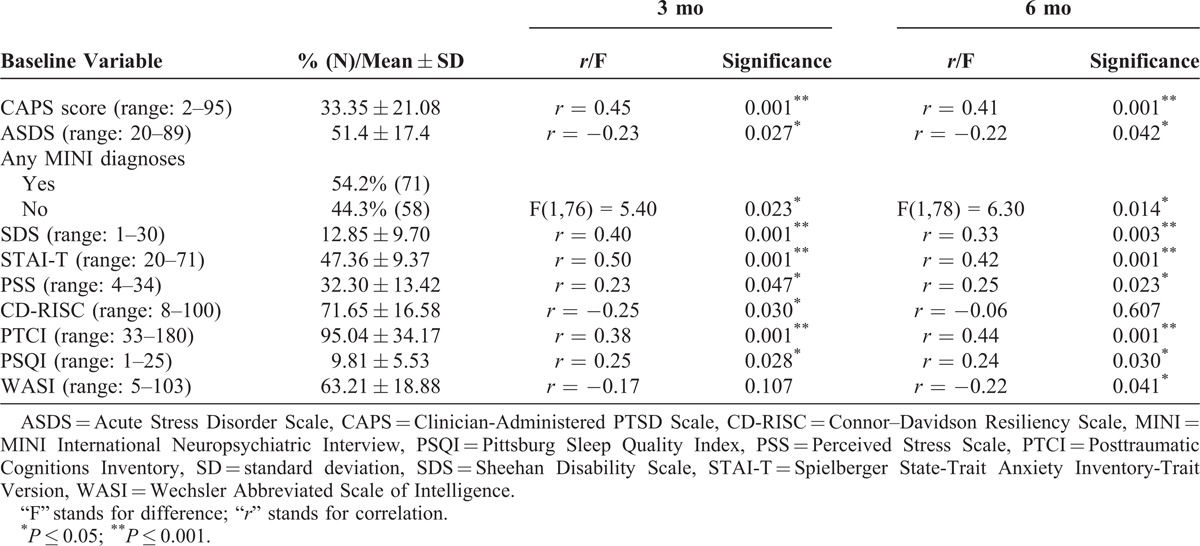
Clinical Characteristics of Sample and Their Relationship to PTSD Severity at 3 and 6 mo Follow-Ups

Three and 6 months after the trauma, baseline disability (SDS), trait anxiety (STAI-T), perceived stress (PSS), negative cognitions (PTCI), sleep disturbances (PSQI), ASD (ASDS), and the presence of any MINI disorder (most commonly depression, dysthymia, generalized anxiety disorder, and risk for suicide) were positively associated with PTSD severity indicating that those with higher scores on these scales were likely to have worse PTSD symptoms. Resilience (CD-RISC) was negatively associated with PTSD severity 3 but not 6 months post-trauma, suggesting that lower resilience was associated with worse symptoms at 3 months.

Potential confounding variables included those that have previously been found to be associated with PTSD and are listed in Table 3. Those that were significantly associated with PTSD severity (*P* < 0.05) or approached significance (*P* < 0.10) were included in the regression analyses. Again, as income and education were significantly related, income was excluded from the analyses. Notably, variables such as age and gender, which have been well established in previous studies as pre-trauma risk factors across trauma type, were not significantly associated with PTSD severity. Variables that were controlled for, therefore, included education and ethnicity at 3 and 6 months.

**TABLE 3:**
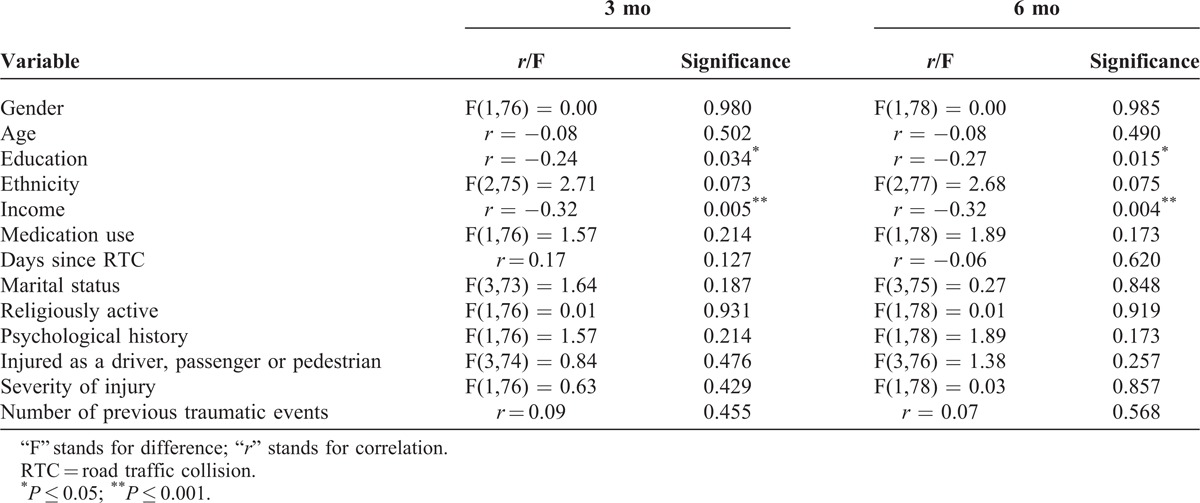
Variables Controlled for at 3 and 6 mo and Their Relationship to PTSD Severity

### Clinical and Neuropsychological Predictors of PTSD

All clinical and neuropsychological predictors and potential confounders of PTSD were included in best subsets regression models to determine those that were most consistently predictive.

The 3-month model was significant and explained approximately 50% of the variance (*R*^2^ = 0.53, adjusted *R*^2^ = 0.46, F(7,48) = 7.73, *P* < 0.001). Six predictors were retained in the model—education, ethnicity, STAI, TMT (A), DSy, and SS. All were significant and were most consistently predictive in the 20 best models too (Table 4).

**TABLE 4:**
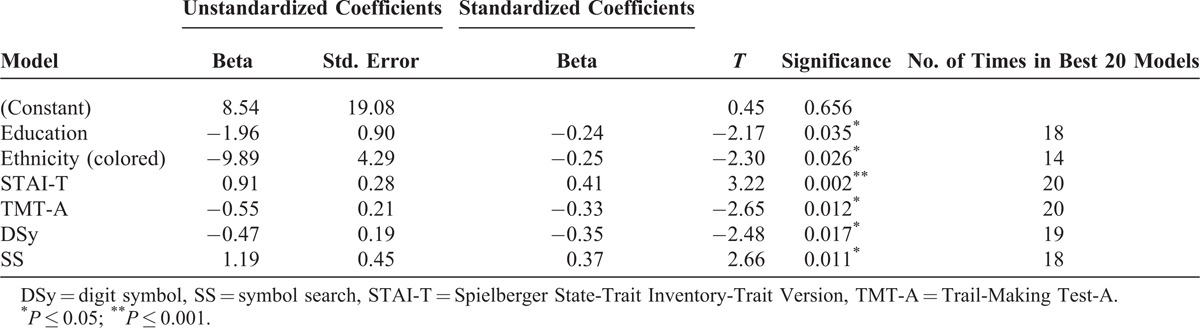
Best Subsets Regression Results for 3-mo Visit

The 6-month model retained 7 predictors—education, ethnicity, STAI, TMT (A), Maze Test, RAVLT-learning score, and VR-delayed recall, which were all significant. The overall model was also significant and explained approximately 40% of the variance (*R*^2^ = 0.48, adjusted *R*^2^ = 0.39, F(7,41) = 5.41, *P* < 0.001). The retained predictors also appeared to be the most consistently predictive variables in the 20 best models (Table 5).

**TABLE 5:**
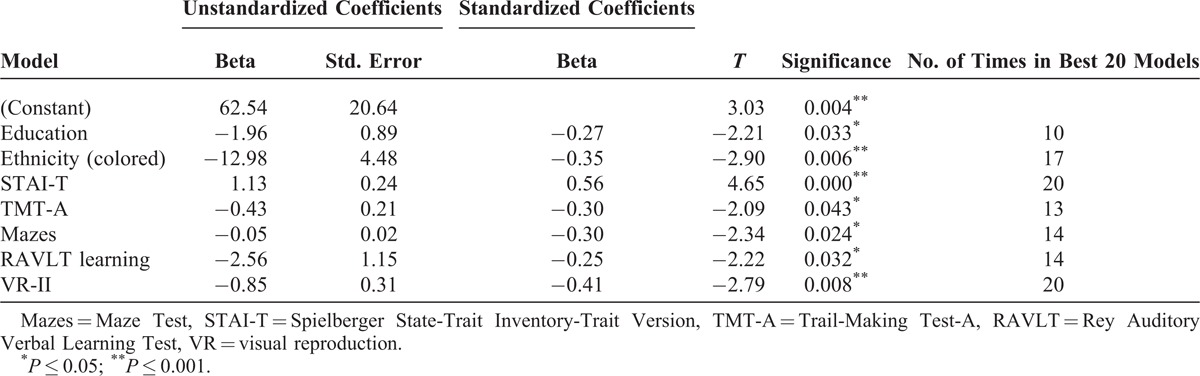
Best Subsets Regression Results for 6-mo Visit

## DISCUSSION

This sample of RTC survivors had moderately severe PTSD symptomatology; 22.9% met criteria for PTSD at baseline (although the duration criterion, >1 month, was not met) and rates of PTSD at 3 and 6 months were 19.6% and 12.2%, respectively. The objective of this study was to extend existing knowledge of early predictors of PTSD severity. In order to do this, we analyzed neuropsychological and clinical variables and their associated sociodemographic factors separately before combining them. We expected baseline sociodemographic, injury characteristics, neuropsychological, and clinical variables to influence vulnerability to PTSD.

Although numerous studies^3,5^ have reported a relationship between PTSD severity and various patient characteristics, we mostly did not find this to be the case. We, however, did find that lower levels of education and ethnicity were associated with follow-up PTSD severity. Education also appears to be one of the most consistent predictors of PTSD severity. It may be that lower educational levels are associated with dysfunctional cognitive and coping styles. Ethnicity too appears to be useful in predicting PTSD in both the short and longer terms, with “colored” ethnicity seeming to be protective. Although this finding is in contrast to a systematic review of factors that predict PTSD after RTCs, which found that ethnicity had no utility in predicting PTSD,^5^ there are some studies that have found ethnicity to relate to the trauma response.^38^

Cognitive changes resulting from trauma exposure or preexisting maladaptive cognitive styles may play a critical role in the development of PTSD.^39,40^ Furthermore, memories of the traumatic event and interpretations of one’s PTSD symptoms or the consequences of the traumatic event may become associated with automatically activated negative evaluations.^41,42^ Although all our clinical variables were found to be associated with PTSD severity 3 and 6 months post-RTC (with the exception of resilience that was only associated with PTSD severity at 3 months post-trauma), trait anxiety was the only one found to predict PTSD severity. In line with our previous findings of an association between trait anxiety and ASD severity,^43^ this suggests that individuals with a predisposition to anxiety problems are prone to developing clinically significant stress responses in relation to life stressors.^44,45^ Thus, it seems that a close examination of temperamental variables, such as the tendency to respond with elevations in anxiety to perceived stressful situations, may lead to better predictions of risk and vulnerability associated with PTSD. In this study, it was the best and most consistent predictor of 3 and 6-month PTSD severity.

Stress-related neurobiological activity is thought to alter the function, and potentially even the structure, of several brain regions mediating the stress response.^46^ Neurobehavioral alterations, in turn, may promote survival by directing focus to potential sources of danger and away from irrelevant aspects of the environment.^47^ Since the ability to simultaneously engage in multiple focused cognitive activities is not unlimited, the diversion of cognitive resources to processing potential threat occurs at the expense of other cognitive activities.^48^ Thus, although this response can result in heightened behavioral reactivity, it can also result in attenuated attention, learning, and memory for nonthreat-relevant stimuli and events.^49,50^

Accordingly, PTSD is associated with significant impairments in cognitive functioning.^51^ In the current study, we found that executive function and information-processing deficits were associated with PTSD severity at both time points (3 months: DSy and ToL tasks; 6 months: DSy, SS, TMT (A and B), and ToL tasks). Poorer verbal learning and motor speed were also associated with 6-month PTSD severity. Furthermore, tasks of information processing (TMT-A, DSy, and SS), executive functioning (Maze Test), verbal learning (RAVLT), and delayed visual memory (VR) were all significantly predictive of later PTSD severity.

PTSD has been associated with poor speed, accuracy, and depth of information processing.^52–54^ Cognitive slowing may be attributable to reduced attentional resources,^55^ which could be a consequence of brain resources being directed toward coping with psychological distress, unpleasant internal experiences,^56^ or potential threats in the environment (ie, hypervigilance) rather than the task needing attention.

Verbal and visual memory deficits, as well as immediate and delayed memory, have previously been found to be predictive of PTSD.^11–13,17^ This suggests that more limited verbal abilities, specifically with regard to acquisition and encoding,^54,57,58^ and difficulty retaining newly learned visual information—whether inherited or a response to life stresses—may increase the risk of PTSD in individuals exposed to trauma while enhanced verbal abilities may act as a protective factor.^59^

Sustained attention^12^ and working memory^13^ have been found to predict PTSD. We did not find that tests of working memory, that is, DS, were predictive of PTSD severity, but we did find more complex tasks of planning and foresight, that is, the Maze Test to be predictive of longer-term PTSD. This suggests that impairment is more likely to be detected on tests of more complex executive function abilities,^60^ and points toward impairments in executive function that relate to the severity of PTSD symptoms, and may serve as a risk factor for the development of the disorder,^7^ for example, by reducing the individual’s capacity to suppress intrusions.^61^ Abnormalities in executive functioning might reflect frontal–subcortical impairment,^53^ which possibly also contributes to hallmark symptoms of PTSD, such as heightened arousal and re-experiencing symptoms and the reliance on avoidant coping strategies.^7^

The primary aims were to identify whether neurocognitive factors are useful in the temporal prediction of PTSD severity and examine the predictive power afforded by their interaction with clinical, sociodemographic, and RTC-related variables. We hypothesized that these variables would significantly predict both shorter (3-month PTSD severity) and longer-term (6-month PTSD severity) outcomes. The 3 and 6-month models were both significant, predicting approximately 50% and 40% of the variance, respectively.

We also expected participants with more severe PTSD at 3 and 6 months to significantly demonstrate more cognitive impairments than participants with fewer symptoms. Although not true of all neurocognitive tests, tests of information processing, executive functioning, verbal learning, and motor speed were related to PTSD severity on univariate analyses, as well as when neuropsychological, clinical, and sociodemographic factors were all taken into account (with the exception of motor speed).

Finally, we expected that participants with more severe PTSD at 3 and 6 months would have more psychiatric symptoms, such as those associated with anxiety, psychiatric disability, or ASD at 2 weeks post-trauma. All clinical variables assessed (ASD, PTSD symptoms, other psychiatric diagnoses, disability, trait anxiety, perceived stress, negative cognitions, and sleep) were associated with 3 and 6-month PTSD severity on univariate analyses, with the exception of resilience, which was only associated with 3-month PTSD severity. Of these, trait anxiety was the only clinical variable found to be predictive of PTSD severity and was consistently the best predictor.

These findings are limited by a number of factors. These data were collected prior to the development of PTSD, but subsequent to a traumatic event, future studies would benefit from the inclusion of participants prior to trauma. The sample consisted of individuals who had experienced a RTC. Although this avoids potential trauma-related confounders, the generalizability of findings is limited. The possibility that medical and surgical factors (ie, physical injury and medication use) may have influenced clinical and cognitive responses cannot be excluded. However, as we controlled for potential confounders where bivariate relationships were observed, these are unlikely to account for the relationships we did find. Furthermore, a larger sample size would have resulted in increased power to detect predictors.

These limitations notwithstanding, the present data offer preliminary evidence for the role of acute neuropsychological, clinical symptomatology and sociodemographic variables in the temporal prediction of PTSD, in a South African sample of RTC survivors. The strengths of the study include the longitudinal nature, reliable measures used in assessment of PTSD, ASD, and other psychiatric disorders, a wide range of possible predictors that were included, and potential confounders that were controlled for.

These findings will hopefully lead to a better understanding of the development and early maintenance of PTSD and serve as a starting point for the development of a screening measure to identify individuals at increased risk of PTSD. This would allow for targeted interventions to treat early negative symptoms and prevent the development of longer-term negative psychiatric sequelae. Furthermore, understanding the acute trauma response may aid in the development of more focused interventions that can reverse early PTSD or prevent the development of the disorder. Recent research indicates that attention modification programs, for example, may be beneficial in anxiety disorders,^62,63^ suggesting that research related to attention and working memory function may not only increase our understanding of PTSD but also lead to more effective treatments.^7^ Implications of these findings therefore suggest the inclusion of neuropsychological measures in the screening of PTSD symptoms, and support a need for treatments that address neurocognitive variables, as well as cognitive and emotional adaptation strategies in the maintenance of PTSD.

In conclusion, this study shows that a combination of neuropsychological, clinical, and sociodemographic characteristics have the best predictive value for PTSD severity in this sample, supporting the need for interventions that target these variables. Given that PTSD can have profound effects on patients and society, early, targeted profiling of this group of trauma survivors can inform early clinical interventions and policy.

## ACKNOWLEDGMENT

The authors would like to thank Prof Kidd (who agrees to be named), Department of Statistical Sciences, Stellenbosch University, Cape Town, South Africa, for his assistance with statistical analyses.
